# Graphene-Based Composites as Catalysts for the Degradation of Pharmaceuticals

**DOI:** 10.3390/ijerph18041529

**Published:** 2021-02-05

**Authors:** Olalekan C. Olatunde, Damian C. Onwudiwe

**Affiliations:** 1 Material Science Innovation and Modelling (MaSIM) Research Focus Area, Faculty of Natural and Agricultural Sciences, Mafikeng Campus, North-West University, Private Bag X2046, Mmabatho 2735, South Africa; Olalekan.olatunde@eksu.edu.ng; 2 Department of Chemistry, School of Physical and Chemical Sciences, Faculty of Natural and Agricultural Sciences, Mafikeng Campus, North-West University, Private Bag X2046, Mmabatho 2735, South Africa

**Keywords:** pharmaceuticals, advanced oxidation process, graphene, composites, reactive radicals

## Abstract

The incessant release of pharmaceuticals into the aquatic environment continues to be a subject of increasing concern. This is because of the growing demand for potable water sources and the potential health hazards which these pollutants pose to aquatic animals and humans. The inability of conventional water treatment systems to remove these compounds creates the need for new treatment systems in order to deal with these class of compounds. This review focuses on advanced oxidation processes that employ graphene-based composites as catalysts for the degradation of pharmaceuticals. These composites have been identified to possess enhanced catalytic activity due to increased surface area and reduced charge carrier recombination. The techniques employed in synthesizing these composites have been explored and five different advanced oxidation processes—direct degradation process, chemical oxidation process, photocatalysis, electrocatalyis processes and sonocatalytic/sono-photocatalytic processes—have been studied in terms of their enhanced catalytic activity. Finally, a comparative analysis of the processes that employ graphene-based composites was done in terms of process efficiency, reaction rate, mineralization efficiency and time required to achieve 90% degradation.

## 1. Introduction

**Figure 1:**
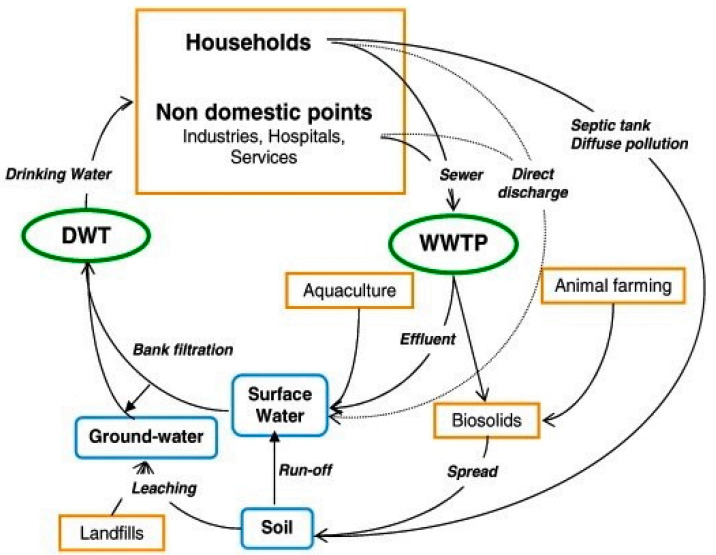
Origins of pharmaceuticals and their environmental routes (WWTP—wastewater treatment plant; DWT—drinking water treatment). Reprinted form Sayadi, et al. [20].

Pharmaceuticals are compounds that are produced for the purpose of usage as medicinal drugs. They remain however as persistent, biologically active compounds which could be detected in drinking and surface waters due to their incomplete removal by conventional processes [1]. These materials have received much attention recently due to their health and environmental implication [2,3,4]. The design of pharmaceuticals is such that when taken even at low concentrations, they can effect physiological changes in humans and animals. These compounds, when excreted either as metabolites or in an unchanged state, remain in the environment for a long time because they are not biologically degraded or reduced [5,6]. In aquatic environments, direct discharge of untreated and treated wastewater from treatment plants are the major source of pharmaceuticals, while terrestrial runoff from agricultural fields, aquaculture facilities and livestock farms are some of the secondary sources as shown in Figure 1 [7,8]. Aquatic channels act as transport routes for pharmaceuticals and their bioaccumulation is mainly due to their lipophilic character, although other factors such as animal homeostasis, metabolism and inhalation exposure are also important in understanding their accumulation in organisms [9,10,11,12]. Compared to other organic contaminants, hydrophobic interactions are not the main influencing parameter on the partitioning dynamics of most pharmaceuticals. Other factors including cation exchange, surface complexation, hydrogen bonding and cation bridging also play vital roles. Thus, modelling approaches that have been created for other organic contaminants such as persistent organic pollutants are inappropriate for assessing the environmental risks of pharmaceuticals [13].

The increasing surge in the global consumption of pharmaceuticals with the global pharmaceutical market worth expected to reach $1170 billion in 2021 from $935 billion in 2017. This implies that concerns over the pollution of the environment by the discharge of different types of pharmaceuticals will continue to grow. This account for them being tagged as part of the emerging anthropogenic hazard pollutants [14,15]. Also, their presence in the environment is due mainly to the inability of treatment plants to completely eliminate them. This, consequently, has led to their detection at concentration range of ng/L to μg/L in surface water, tap water and ground water [16,17,18]. The increase in the concentration of pharmaceuticals in the water system, implies that the reuse of treated water, which has been suggested as the adequate solution for the sustainability of water resource is also under serious treat [19].

Pharmaceuticals and their by-products could be classified into 24 therapeutic classes, with anti-inflammatory drugs (NSAIDs) (e.g., ibuprofen, gemfibrozil and diclofenac), antibiotics (e.g., sulfamethoxazole and trimethoprim), anticonvulsants (e.g., carbamazepine) and lipid regulators (propranolol, metoprolol, nadolol) being the four most studied categories. Increasing information on the potential chronic or acute effects of pharmaceuticals on the ecosystem and living organisms have begun to emerge recently. Estrogen acts as endocrine modulators or disruptors and can have adverse effect on reproductive and sexual development like feminization of male fishes even at ng/L level [18,21]. Diclofenac has been indicted for its effect on mammalian kidney and the disappearance of the Oriental White backed Vulture in India and Pakistan [22]. The increased resistance to antimicrobial agents is another risk associated with the presence of pharmaceuticals in the environment [23]. Table 1 presents some therapeutic classes of pharmaceuticals with their acute toxicity level and concentrations reported in the environment.

**Figure 2:**
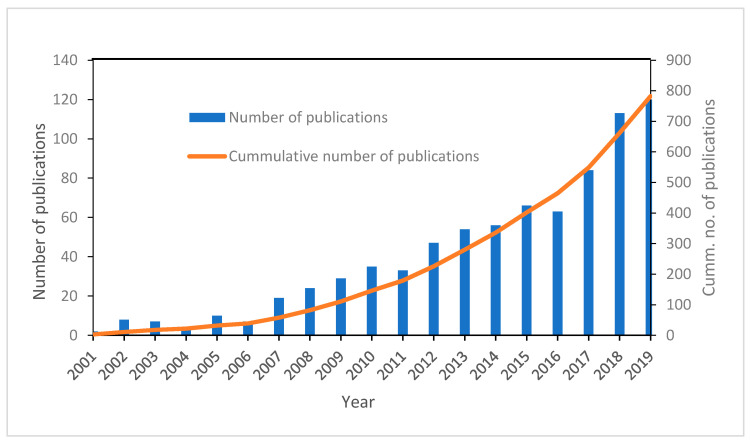
Timeline distribution of the annual and cumulative number of publications on the use of advanced oxidation processes for the degradation of pharmaceuticals.

Since conventional water and wastewater treatment processes (WWTP) are incapable of completely removing pharmaceuticals, it is therefore important for advanced treatment techniques to be incorporated into WWTPs. Some of the advanced processes that have been explored for the removal of pharmaceuticals include chemical oxidation processes, membrane technologies and adsorption [36]. Membrane technologies and adsorption process are economical physical techniques, with relative ease of handling; however, these processes are highly material and energy intensive [37] and the problem of membrane retentate and spent adsorbent disposal means there is high possibility of reintroducing the removed contaminants back into the environment. Thus much focus has beenon oxidation processes, in which contaminants are degraded by highly reactive radical species species generated within the system. These processes are referred to as advanced oxidation process (AOPs) and as shown in Figure 2, there is a continual growth in the body of literature on their application in pharmaceutical degradation. Advanced oxidation process is an aqueous phase oxidation technique that is based on the action of highly reactive species such as hydroxyl radical ^(•^OH), superoxide anion radical ($O_{2}^{•\;-})$, singlet oxygen ($O_{2}^{•})$ and hydrogen peroxide $\left(H_{2}O_{2}\right)$ [38]. Contaminants’ degradation in AOPs are stimulated either through radiolysis, sonolysis or photolysis. While radiolysis and sonolysis generate radicals in aqueous media without the use of chemical oxidants, photolysis may require the involvement of a catalyst or precursor. AOPs is comprised of a large range of technologies such as electrolysis, Fenton and photo-Fenton processes, wet air oxidation, ionization radiation, pulsed plasma, microwave, chemical oxidation processes, UV-based processes, supercritical water oxidation, and photocatalytic redox processes [39,40]. Also, hybrid AOPs involving the combination of two or more processes such electro-Fenton, and photo-Fenton processes have also been explored for the degradation of contaminants.

Advanced oxidation processes have generated a lot of attention because of their rapid degradation rates for a wide range of chemicals, the ability to convert pollutants into simpler and biodegradable forms and the potential for complete mineralization of most pollutants. In addition, there is no need for secondary treatment of residual solids since no residual solid is generated and no regeneration is required to sustain process efficiency. Despite these advantages of AOPs, the process suffer from some disadvantages, which include: generation of unknown and undesirable transformation products; process may be capital intensive; water quality significantly impede the efficiency of the process and secondary processes for residual oxidant quenching may be required [41].

The use of heterogeneous photocatalytic process (HPPs), among other advanced oxidation processes, has gained much attention in the past three decades and has proven to be a suitable technique for the degradation and mineralization of different contaminants via a series of multi-step reactions to form low molecular weight end products, such as carboxylic acid, CO_2_ and H_2_O [42,43]. The preference for HPPs is due mainly to their important, characteristic features which include: (i) ambient operating conditions, such as temperature and pressure (ii) potential mineralization of parent and transformation products without secondary pollution and (iii) economic feasibility and efficiency [44]. A broad class of semiconductor nanomaterials have been explored for HPP processes and could be broadly classified as metals, sulfides [45,46], oxides [47,48], nitrides [49,50], oxysulfides [51], oxyhalides [52,53] and oxynitrides [54,55]. The activity of semiconductors in light-induced redox process sensitization arise from the electronic structure of the metal atoms in chemical compounds. The electronic structure of these compounds is made up of filled valence band (VB) and an empty conduction band (CB) [43].

Supporting catalysts on suitable materials provides routes for the improvement of properties such as quantum efficiency, adsorption capacity, electronic band structure, stability, dispensability and the overall reaction rate of the process. Several class of materials currently being explored as catalyst-support include [56]: (i) carbon-based supports e.g., activated carbon, carbon nanotubes, graphite, graphene/graphene derivatives; (ii) ceramic-based supports e.g., silica, alumina, zirconia, zeolite, and silica carbide; (iii) metal-based supports e.g., chromium, platinum and cupper; and (iv) polymeric-based supports e.g., chitosan, polylactic acid, polyester and polyamides. Graphene or graphene derivatives such graphene oxide, reduced graphene oxide and doped graphene oxides are important class of support materials for catalytic degradation of contaminants in water. Incorporation of catalysts into graphene offer advantages such as high surface area and tensile strength, improved mobility and electron conductivity, lightweight, and stability [57].

There are several reviews on the degradation of pharmaceuticals in water [36,58,59,60], with focus mainly on adsorption and photocatalytic processes. In this present review, the degradation activity of graphene and graphene-based composites in AOPs such as direct catalytic degradation, electron-Fenton and chemical oxidation process, and sonocatalytic/sonophotocatalytic processes will be explored in addition to photocatalytic processes. Also, a comparative analysis of the identified processes will be carried out.

## 2. Graphene/Graphene Derivatives

Graphene is regarded as the strongest, thinnest and lightest compound known till date, and these properties are related to its structural features such as interlayer crosslinks arising either from covalent bonding between side atoms of different sheets or Van der Waals forces arising from interactions between carbon atoms of different layers of the graphene sheet. Also, intralayer forces, such as the sp^2^ carbon-carbon covalent bonds and crosslinks occurring at the graphene sheet edges contributes to the unique properties of graphene and its derivatives. Graphene-based materials are therefore outstanding materials for catalyst support in catalysis as they improve the stability and surface area of the catalyst, which is important in bringing the pollutants closer to the catalyst surface where catalytic activities occur.

Perfect graphene has a zero bandgap, and this makes pristine graphene a perfect conductor with high conductivity. The zero E_g_ of graphene is due to the anti-bonding ℼ* orbitals and bonding **ℼ** degenerate that meets at the Brillouin zone corners [61]. Doping of graphene leads to the formation of bandgap between the ℼ* and **ℼ** orbital, thereby converting graphene into a semiconductor and creating more active sites for reactions. Large bandgap could be achieved by high dopant concentration, thus improving the electrical property of graphene [62,63].

**Figure 3:**
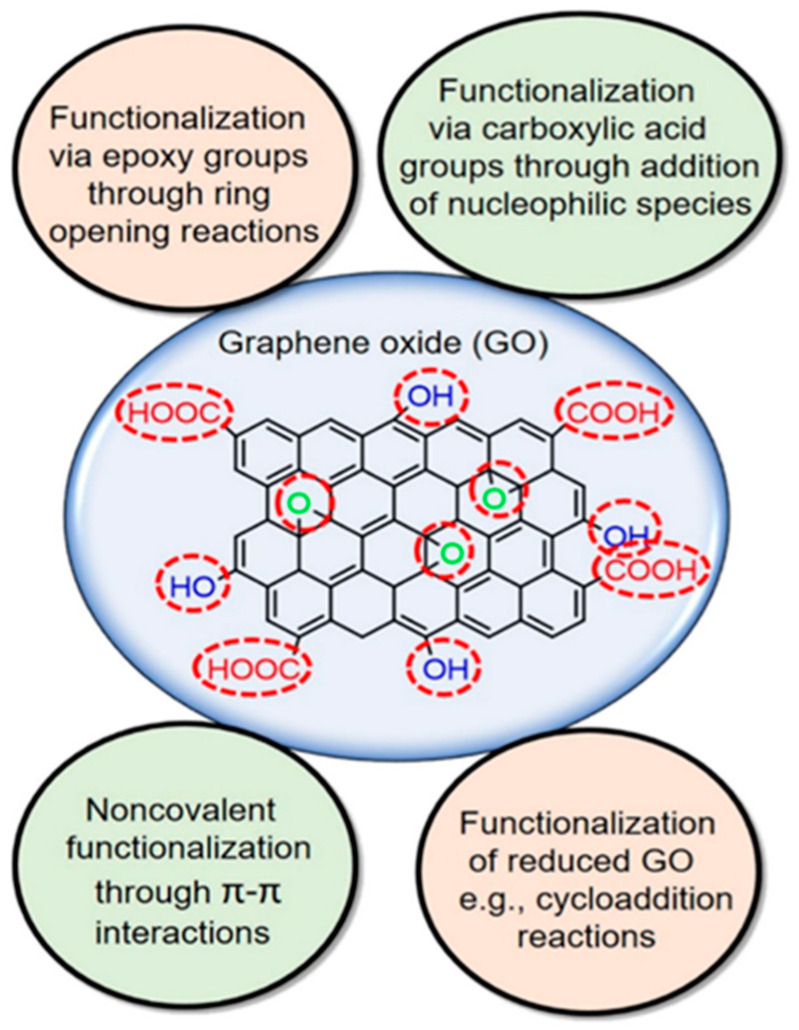
Different routes to graphene/graphene derivatives functionalization. Reproduced from Bilal, et al. [70]. Copyright (2020) Elsevier.

Electron arrangement in the graphene lattice is dominated by the s and p orbitals. However, the low dispersibility, difficult recovery and ease of restacking are major drawbacks to its application. This informed the development of many strategies to modify it by incorporating several functional groups such as halogens, chalcogens, boron, nitrogen and phosphorus [64]. Complex functional groups such as aryl and alkyl hydrocarbons have also been reportedly incorporated [65]. The methods of incorporating functional groups into graphene are shown in Figure 3. The most studied functionalized graphenes are graphene oxide (GO) and reduced graphene oxides (rGO), which are derivatives with poorly defined compositions that are strongly influenced by the synthesis method [64]. The abundance amount of oxygen functionalities on the GO surface, coupled with other properties such as structural defects, irregularity of heteroatoms makes them potential materials for specific applications. In graphene oxide, the electronic properties is strongly determined by its chemical structure [66]. Compared to graphene, GO is hydrophilic and an insulator by nature because of the presence of numerous surface hydroxyl (−OH), keto (C=O), epoxy (C-O-C) and carboxy (−COOH) groups [67]. The formation of C-O bond significantly induces the local distortion of graphene, altering the bonding character from planar sp^2^ to partial sp^3^ hybridization. By reducing GO, partial restoration of the intrinsic structural and electronic properties of graphene can be restored to produce rGO. However, the removal of oxygen-containing functionalities can lead to the rapid re-aggregation of rGO nanosheets into graphite [68]. Considering an arrangement of epoxy functional groups in fully oxidized graphene sheet and the effect of epoxy arrangement on electronic properties, a significant induction of bandgap of 0.529 eV has been reported [69].

### 2.1. Synthesis of Graphene Oxide and Reduced Graphene Oxide

GO is widely employed in several applications because it is easy to synthesize, process and functionalize chemically. It is generally prepared either by the Brodie, Staudenmaier, Hummers, Tours or by modified forms of any of these methods. All the methods involve the oxidation of graphite to varying levels. The wet-chemical oxidative process used to convert graphite into GO involves four steps: (i) intercalation of graphite, (ii) oxidation, (iii) oxidized sheets exfoliation, and (iv) impurity removal [71,72,73,74,75,76]. The intercalation step involves the insertion of small molecules within the graphite layers. Alkali compounds such as NaNO_3_ and KClO_3_ are usually intercalated into the graphite layers, which is then subsequently oxidized by acids such as HNO_3_, HCl and H_2_SO_4_. The properties of the final product are influenced by several factors such as graphite’s nature and source, nature and concentration of hydrogen peroxide, oxidant, water and sulfuric acid and physical parameters such as reaction time and temperature. The first reliable method for GO synthesis from graphite was reported in 1855 [77]. It involves the treatment of graphite with fuming HNO_3_ and KClO_3_. This method was improved on by using H_2_SO_4_ as additive and also the use of excess HNO_3_ [78]. It gave highly oxidized GO ‘within a shorter period of time’ compared to the Brodie method. The widely used Hummer’s method involves the treatment of graphite with NaNO_3_, KMnO_4_ and H_2_SO_4_. Recently, the Tour’s method as described by Marcano, et al. [79], introduced the use of H_3_PO_4_ as co-acids with H_2_SO_4_ and six equivalents of KMnO_4_ as oxidant.

**Figure 4:**
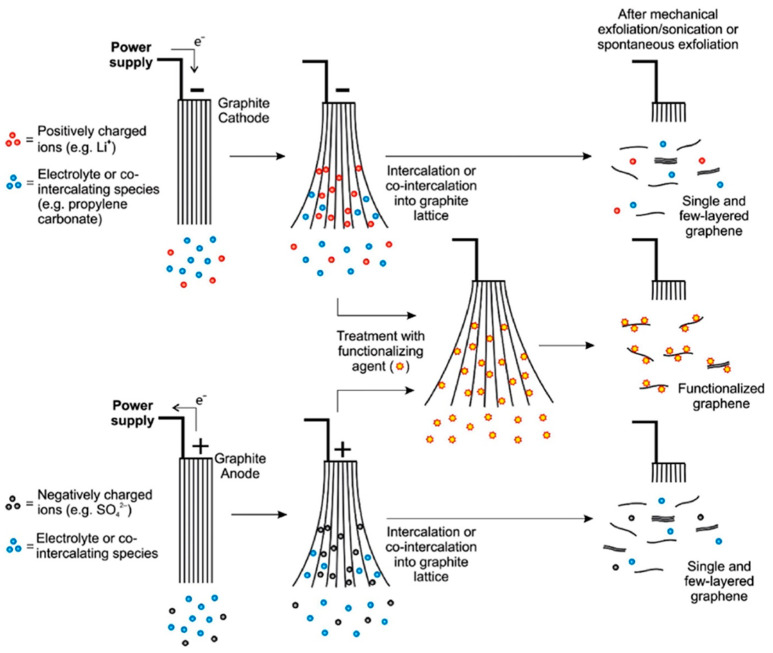
A schematic overview of mechanism of cathodic and anodic exfoliation. Functionalization of graphene may be achieved either during exfoliation by introducing functionalizing agent or after exfoliation. Reprinted with permission from Yu, et al. [83]. Copyright (2015) Elsevier.

Electrochemical stripping of graphite is another well explored technique for GO synthesis. This method is a safe, pollution-free, and efficient technique involving no chemical oxidant and achievable within hours or sometimes minutes. It also offers ease of manipulating GO properties such as defect density and degree of oxidation by tuning the electrical process [80,81]. The technique explores the conductivity of graphite to intercalate anions/molecules in the electrolyte under bias current or voltage. The GO is then obtained by ultrasonication of the graphite oxide formed through the oxidation of the graphite intercalation compound by oxygenated species produced by the electrolysis of water [82]. The mechanism of formation of graphene oxide via the electrochemical stripping process is shown in Figure 4. The mechanism could either be anodic or cathodic based on the applied potential. In anodic process, electrons are withdrawn from the graphite electrode leading to the creation of positive charge, allowing for the intercalation of negatively charged ions like sulfate anions. In cathodic process, negative charges created at the graphite electrode attracts positively charge ions. Incorporation of these ions increase the interlayer spacing between the graphene sheets and enhance its exfoliation [83].

**Figure 5:**
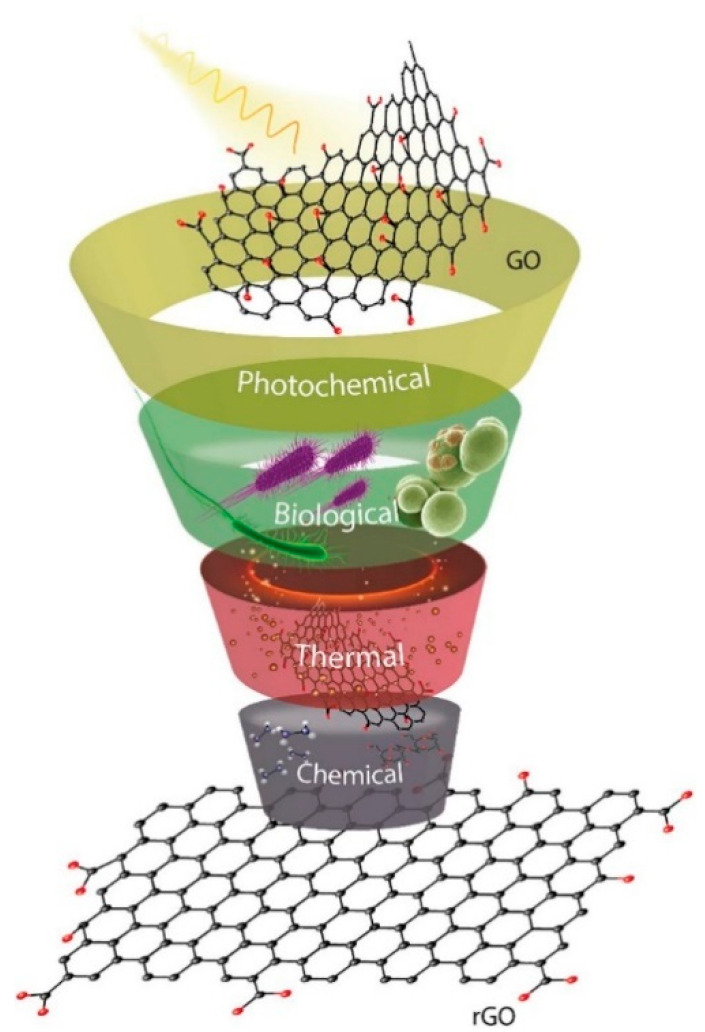
Routes to reduction of graphene oxide. Reprinted with permission from Agarwal and Zetterlund [100]. Copyright (2021) Elsevier.

The removal of oxygen functional groups from GO is a subject of continued research, with different methods such as thermal, photochemical, biological and chemical methods some of the already established routes as depicted in Figure 5 [84]. Each method produces rGO of varying properties such as morphology, dispersibility, conductivity and mechanical strength. The choice of the reduction method is influenced by factors such as C/O ratio of end product, selectivity in removal of either −OH, −COOH or C-O-C functionalities, choice of reducing agent, surface defect reduction and improvement in chemical and physical properties [85]. The chemical method is the most explored routes to rGO and it involves treating GO suspension with reducing agents such as hydrazine [86,87,88], ascorbic acid [89,90,91,92], sodium borohydride [93,94,95,96] and sodium hypophosphite monohydrate [97,98]. In a report by Luo, et al. [99], the reduction of GO by N_2_H_4_•H_2_O, NaBH_4_, NaOH, solvothermal, high-temperature, and a two-step method combining NaBH_4_ and high temperature was comparatively studied using four criteria: degree of reduction, level of defect repair, dispersibility, and electrical conductivity. The study showed that by combining two reduction processes, the properties of rGO could be greatly enhanced.

**Figure 6:**
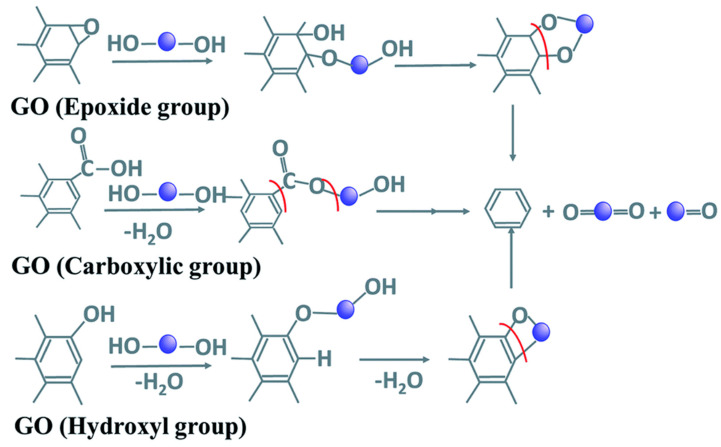
Reduction of oxygen functionality in GO by plant extracts. Adapted with permission from Bhattacharya, et al. [101] under Creative Commons Attribution 4.0 International.

Recently, the use of plant extracts as green reductants for rGO synthesis has been studied. The method involves the use of extracts from different plant parts such as seeds, roots, flowers and fruits. The reduction could be initiated by polyphenols (such as catechol and pyrogallol) or flavonoids (glucoside, diosmetin and apigenin) in the plant extracts. According to the mechanism proposed by Bhattacharya, et al. [101], shown in Figure 6, the carboxylic groups in GO reacts with reduced species in plant extracts to form esters by condensation reaction, which is further reduced by ring opening reaction. The hydroxyl groups are reduced by undergoing an initial condensation reaction, which is also followed by ring cleavage, while epoxy group reacts with polyhydroxy groups in extracts to form an intermediate that undergoes subsequent ring formation and cleavage.

### 2.2. Composites of Graphene/Graphene Derivatives

Increased utilization of graphene and its derivatives have motivated the exploration of different functionalization routes. Compositing of graphene with other nanomaterials take advantage of its superior properties to enhance catalytic activities. Graphene possess a two-dimensional carbon network with sp^2^ hybridization and it exhibits unique properties including high specific surface area, high intrinsic electron mobility and high thermal conductivity [102]. High degradation of pollutants could be achieved by hybridizing graphene with catalysts due to its effective electron conductivity and effective minimization of charge carrier’s recombination via its acceptance of photogenerated electrons.

**Figure 7:**
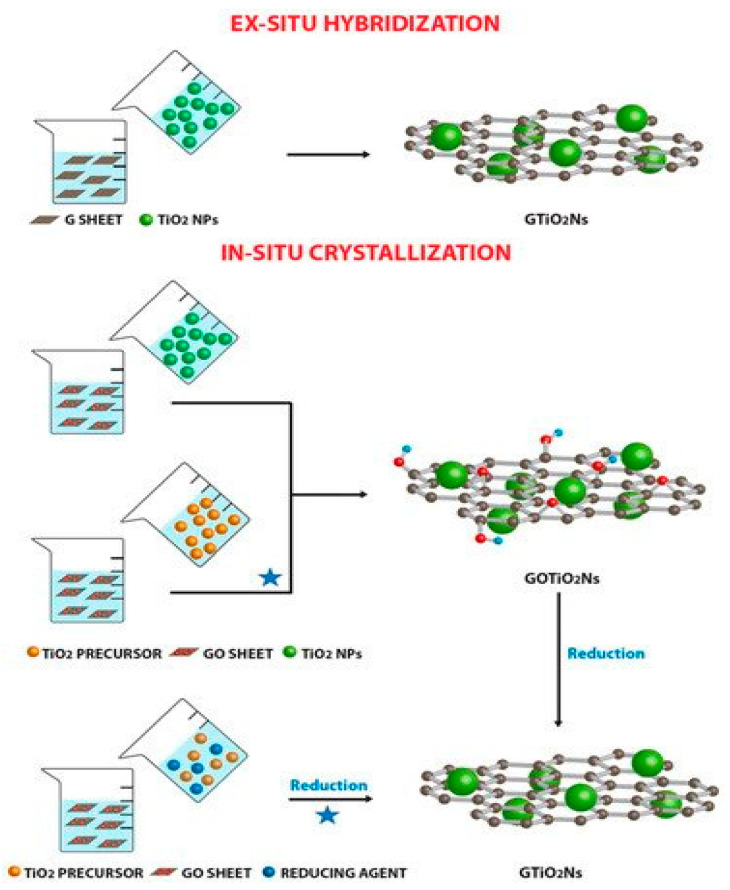
In-situ and Ex-situ compositing of metal salt with graphene and graphene derivatives. Reproduced with permission from Giovannetti, et al. [108] under Creative Commons Attribution 4.0 International.

The improvement of catalytic activity by graphene and its derivatives is mainly due to their ability to extent light absorption range, absorptivity enhancement, enhanced surface area and improved charge separation and transport [103]. The compositing of a photocatalyst results in decreased h^+^/e^−^ recombination and enhances the electron transfer rate, while also enhancing chemical species adsorption via **ℼ**-**ℼ** interactions [104]. Generally, in graphene-based composites, graphene can either act as a support [105], dopant or a coating [106,107]. The three classes of composite could be prepared either via “ex situ” or “in situ” compositing as shown in Figure 7.

Ex situ compositing process involves the mixing of nanoparticles with graphene dispersions. To enhance the process, the graphene sheets may be functionalized through covalent C-C coupling or non-covalent **ℼ**-**ℼ** stacking reactions [109]. Ex situ compositing may sometimes result in low density and non-uniform distribution of the nanoparticles on the graphene. Several synthetic routes have been developed based on the ex situ process. AbdelDayem, et al. [110] reported the ex situ compositing of alumina with graphene oxide by mixing pre-prepared alumina with GO solution and refluxing the mixture at 80 °C for 8 h. A two-phase method to the compositing of GO and CdS was reported by Gao, et al. [111]. In this method, synthesized GO and CdS were dispersed in water and toluene respectively. The two solutions were then mixed together and stirred continuously for 24 h, followed by washing and drying. The interaction between the GO functional groups and CdS led to the distribution of the CdS on the GO surface and the aggregation of the CdS particles was minimized by the process. The synthesis of TiO_2_@rGO through mechanochemical synthesis involving the grinding of TiO_2_ nanoparticles and rGO was also reported by Deng, et al. [112].

The in-situ method is the most explored for graphene/nanoparticle composites and it involves direct reaction of GO/rGO sheets and nanoparticles’ precursors in solution. The growth of nanoparticles on the surface of GO/rGO have been explored widely through solvothermal and/or hydrothermal techniques. It has been explored frequently in the synthesis of GO/rGO-metal oxide/sulphide nanocomposites. In the in-situ method, nanoparticle nucleation and growth occur on the GO/rGO surface under high-temperature and pressure, with the hydroxyl and carboxyl functional groups acting as nucleation sites for nanoparticles growth via metal-O-C bond formation [113,114,115]. A microwave assisted reduction synthetic method via a mixture of PdCl_2_, RuCl_3_ and GO, was employed for the synthesis of bimetallic-graphene composite. Reduction of the metal salts to metallic state was achieved with NaBH_4_, which was enhanced by holding the mixture in a microwave oven [116]. A method involving the mixture of GO/rGO precursor and nanoparticle precursors was described by Anand, et al. [117] for the synthesis of rGO/Bi_2_Al_4_O_9_. In the synthesis [Bi(NO_3_)•5H_2_O] and AlNO_3_•9H_2_O were dissolved into the graphene oxide synthesis set-up. The mixture was then stirred for 30 min and further sonicated for 5 min. The process of stirring and sonicating was repeated for 4 h, and the reaction was quenched afterwards. GO reduction to rGO was achieved by adding 10% ascorbic acid solution to the reaction system and heating at 95 °C for 1 h. Cao, et al. [118] reported the combination of precursor mixing and annealing for the in-situ synthesis of ZnO/rGO. In this synthesis route, ZnO powder was firstly mixed with GO solution and the mixture stirred vigorously for 3 h. The mixture was further kept at room temperature for 3 h and the product was collected by centrifugation, then dried at 60 °C for 12 h. The product was annealed for 2 h at 350 °C at a heating rate of 2 °C/min to obtain the ZnO/rGO.

Electrochemical exfoliation method is another simple and one-step ex-situ technique used in preparing GO/rGO-composites. Ansari and Payami [119] reported the synthesis of magnetic graphene-Fe_2_O_3_ nanocomposite using this method. The electrolytic cell was made with graphite foil anode and iron plate as cathode, and applying a voltage of 10 V DC for 3 h. In a study to evaluate the effect of synthesis method on the properties of GO/rGO-composite, Aquino, et al. [120] reported the synthesis of GO/WO_3_NW/PANI through chemical and electrochemical routes. It was reported that the electrochemical process resulted in a composite with disorganized structure, which however, increased the doping level in the polymer chain, enhanced porosity and also permitted higher synergistic effect among the components of the composites, when compared to the nanocomposites prepared via the chemical route.

The growth of nanoparticles on GO/rGO sheets is influenced by the amount of functional group present on the sheet [121]. So, compared to rGO, GO can facilitate the growth of nanoparticles leading to higher density and smaller nanoparticle size [122]. Therefore, to overcome low particle loading and poor solubility of rGO, surfactant molecules or polymers are employed in composite synthesis. The synthesis of Pd-CuO/rGO and Au-CuO/rGO via an ionic liquid-assisted approach was reported by Alhumaimess, et al. [123]. The addition of little amount of the ionic liquid 1-butyl-3methyl imidazolium tetrafluoroborate resulted in uniformly dispersed nanoparticles, with simultaneous reduction of GO.

Graphene doped with heteroatoms such as nitrogen (N), sulphur (S), boron (B) have also been explored in synthesis of composites with semiconductors. Ex-situ synthesis of doped-GO/rGO-composite such as N-graphene, and B-graphene have also been reported. Liu, et al. [124] reported the solvothermal synthesis of NG/TiO_2_ by mixing N-graphene oxide and TiO_2_ in a Teflon-lined reactor. The synthesis of Co_3_Sn_2_@Co-NG was also reported by Mahmood, et al. [125] via the in-situ approach.

## 3. Catalytic Activity of Graphene-Based Composites in the Degradation of Pharmaceuticals

The enhanced catalytic properties of graphene-supported composites have been studied in different technologies for the degradation of pharmaceuticals. While the photocatalytic process is the most explored technique, other degradation processes such as direct degradation, chemical oxidation, electrochemical and sonochemical/sono-photochemical processes have also been identified; thus, affirming the versatility of this composites as catalysts for pharmaceutical degradation.

### 3.1. Direct Degradation Processes

Although, most catalytic application of graphene composites in degradation of pollutants have focused on processes involving the addition of chemicals such as persulfate or light energy, few processes have been explored without these complementary components. Saroyan, et al. [126] reported the degradation of bisphenol-A by graphite oxide composited with mixed oxidation state manganese oxide. The improved catalytic activity was correlated with the improved adsorption of bisphenol-A unto the composite, which brought the BPA into close proximity with the Mn_3_O_4_. The activity of the composite was optimal at pH of 3, reaching a degradation efficiency of 93%. The degradation of tetracycline by MnO_2_ supported on a three-dimensional graphene was also reported by Song, et al. [127] with efficiency of up to 91% and rate constant of 0.136 μM/min.

### 3.2. Chemical Oxidation Processes

Peroxymonosulfate (PMS)- or persulfate (PS)-based oxidation processes are effective routes to the degradation of recalcitrant and refractory pollutants because of their great oxidation capacity and high selectivity. The activation of persulfate by processes such as heating, ultrasound, UV light, transition metal ion and alkaline conditions leads to the generation of ${\mathrm{SO}}_{4}^{•\;-}$. Due to the recent interest in heterogeneous catalysis, the use of solid catalysts has gained much focus because of their excellent catalytic activity, stability and ease of separation. Solid catalysts based on magnetic spinel-type ferrite materials (MFe_2_O_4_, M =Co, Fe, Ni, Zn, and Cu) can act as heterogeneous Fenton-like catalysts for the activation of PS. Recent studies have shown that the compositing of these catalysts with GO/rGO could enhance the PS activation activity of these heterogeneous catalysts.

**Figure 8:**
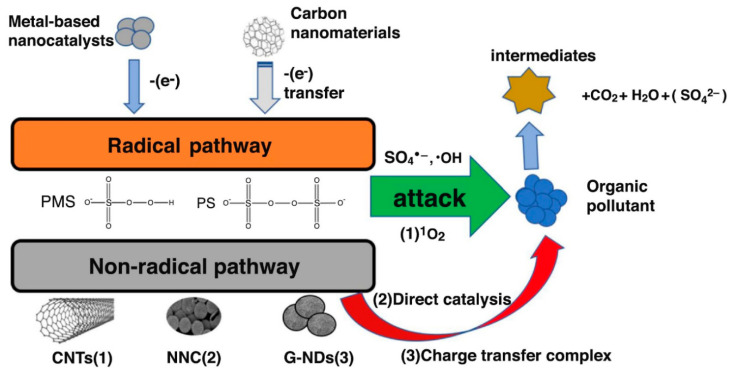
Mechanism of persulfate activation by metal-based catalysts and carbonaceous materials. Reproduced with permission from Xiao, et al. [128]. Copyright (2018) Elsevier.

The primary mechanism of persulfate activation by carbonaceous materials as shown in Figure 8 is based on the one-electron transfer route (Equation (1)) [128,129]. The generation of ^•^OH is also possible via the reaction expressed in Equation (2). In both reactions, radical species generated are responsible for the degradation of organic compounds. Non-radical routes involving transfer of electrons from organic compounds to persulfate and/or singlet oxygen generation could also occur. The ability of doped graphene to effectively activate PS was reported by Chen and Carroll [130] in which 99.9% degradation of sulfamethoxazole was achieved by N-doped graphene. The enhanced activity was attributed to the increased nitrogen groups such as pyridinic-N and quaternary-N, which acts as adsorption sites and source of free-flowing **ℼ**-electrons for PS activation.

(1) $$S_{2}O_{8}^{2-}+e^{-}\to {\mathrm{SO}}_{4}^{•-}+{\mathrm{SO}}_{4}^{2-}$$

(2) $${\mathrm{SO}}_{4}^{•-}+{\mathrm{OH}}^{-}\to {\mathrm{SO}}_{4}^{2-}+{}^{•}OH$$

The degradation of methylparaben by CuFe_2_O_4_-rGO in the presence of PS showed a degradation efficiency of 95% with mineralization efficiency of 73% compared to 38 and 20% degradation and mineralization efficiencies respectively observed for CuFe_2_O_4_. The reaction rate constant for the rGO supported catalyst was almost 7-fold the value observed for bare CuFe_2_O_4_. The improved activity of the supported catalyst was attributed to the direct activation of PS via electron transfer reactions involving the -COOH and O-C=O groups on the rGO surface [131]. The activation of PS by Ni_2_SnO_4_-rGO for degradation of bisphenol A was reported by Jiang et al. [132]. Compositing of the catalyst with rGO reportedly increased the degradation efficiency of the process by 32%. This was attributed to the ability of rGO to prevent the aggregation of the Ni_2_SnO_4_ nanoparticles; thus, increasing the active sites of the catalyst.

**Figure 9:**
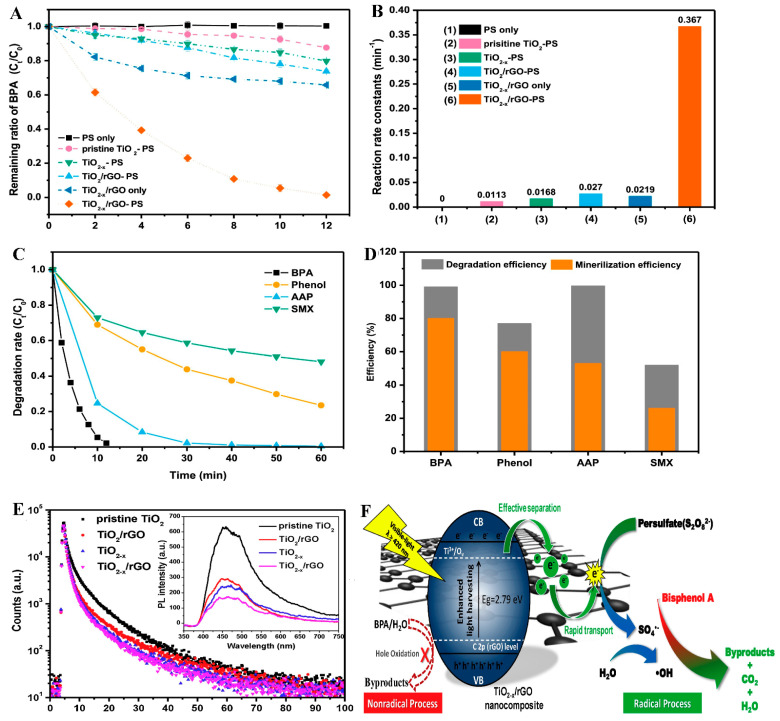
Degradation of bisphenol, phenol, acetaminophen and sulfamethoxazole by TiO_2−x_/rGO (**A**) degradation efficiency of bisphenol by PS only, pristine TiO_2_ +PS, TiO_2−x_ + PS, TiO_2−x_ + PS, TiO_2−x_/rGO, TiO_2−x_/rGO + PS (**B**) kinetic of degradation of bisphenol by PS only, pristine TiO_2_ + PS, TiO_2−x_ + PS, TiO_2−x_ + PS, TiO_2−x_/rGO, TiO_2−x_/rGO + PS (**C**) Efficiency of TiO_2−x_/rGO + PS for bisphenol, phenol, acetaminophen and sulfamethoxazole degradation (**D**) degradation and mineralization of TiO_2−x_/rGO + PS for bisphenol, phenol, acetaminophen and sulfamethoxazole degradation (**E**) Time resolved PL spectra of TiO_2−x_/rGO (inset) steady state PL spectra. (**F**) Mechanism of action of TiO_2−x_/rGO for bisphenol degradation. Adapted with permission from Yang, et al. [134].

Vieira et al. [133] reported the synthesis of a membrane based on carbon-polymer nanocomposite obtained by compositing nitrogen-doped reduced graphene oxide with poly(vinylidene fluoride) (rGO-M-PVDF). The composite was explored for PS activation in the degradation of ofloxacin (OFX), ciprofloxacin (CIP) and enrofloxacin (EFX), with degradation efficiencies of 54, 77 and 91% respectively. The degradation pathway was observed to be by both radical and non-radical oxidation routes. Evaluation of the surface group present in the spent membrane showed that a loss in the N-pyridinic group was observed, while the N-pyrrolic and N-quaternary group remained unchanged in the membrane. This confirmed that the N-pyridinic group accounts for the catalytic activity of the membrane. Also, the non-radical process occurred via the singlet oxygen generated in the reaction system. The degradation of bisphenol, phenol, acetaminophen and sulfamethoxazole by visible-light activated persulfate using Ti^3+^ self-doped TiO_2_/GO composite was reported by Yang, et al. [134]. The efficiency of the process reached 99% after just 12 min for bisphenol degradation, implying the enhanced activation of persulfate by the TiO_2-x_/rGO composite. As shown in Figure 9A,B, the synergy between the self-doped TiO_2_ and rGO was important for enhanced degradation efficiency and high reaction rate. The process showed higher degradation and mineralization efficiency towards bisphenol and acetaminophen, with almost complete degradation achieved as shown in Figure 9C,D. Studies carried out on the degradation process showed that persulfate was activated by electrons generated by the photocatalyst, leading to the generation of ^•^OH and ${\mathrm{SO}}_{4}^{•-}$, which attacks the contaminants as shown in Figure 9F. Photoluminescence spectra of the composite (Figure 9E) showed a significant reduction in e−/h+ recombination, which accounts for high efficiency of the process.

The mechanism of PMS activation by rGO-CoFe_2_O_4_ for ofloxacin and cefazolin degradation was reported by Fan et al. [135]. The study showed that while sulfate radicals were the major active species in ofloxacin degradation, non-radical oxidation by PMS direct two-electron transfer was prevalent in cefazolin degradation. The difference in degradation routes was attributed to the difference in the nucleophilicity and electrophilicity of different sites on the antibiotics. The activation of PMS was achieved via three different redox reactions between Co(II)/Co(III), Fe(II)/Fe(III) and lattice oxygen/O_2_ from rGO-CoFe_2_O_4_. Complete degradation of the pharmaceuticals was achieved under 30 min using 0.1 g/L catalyst concentration and 1 mM PMS. 

Other graphene-based composites employed for the activation of persulfate in pharmaceutical degradation are presented in Table 2. The suitability of this process for pharmaceutical degradation could be observed as high degradation efficiencies and reaction rates were reported for the process. Most of the reported studies were carried out under acidic conditions, thus suggesting the preference for acidic conditions for optimal activity. It is observed that significant degradation of pharmaceuticals could be achieved within relatively short reaction times, which is an important factor for adaptation in treating large volume of wastewater in treatment plants. The presence of chalcogens such as N, O and S on the composite played significant role in persulfate activation and a corresponding increase in the activity of the composites.

### 3.3. Photocatalysis

Photocatalysis involves the use of semiconducting materials such as oxides, sulphides and nitrides, that have the capacity to absorb light energy and generate reactive oxygen species (ROS). The generated ROS such as ^•^OH and $O_{2}^{•\;-}$ are responsible for the degradation of organic pollutants via a redox process [144]. For ROS generation, light of equal or higher energy compared to the band gap of the semiconductor irradiates the semiconductor surface, leading to the excitation of electrons from the valence band into the conduction band and holes are left in the conduction band. This is referred to as electron-hole (e^−^/h^+^) pairs generation. The electronhole pairs react with oxygenated species such as water and air on the surface of the catalyst to generate ROS. The electron is responsible for the formation of $O_{2}^{•\;-}$ via dissolved oxygen reduction, while oxidation of water by hole leads to the formation of hydrogen gas and ^•^OH. The $O_{2}^{•\;-}$ could subsequently produce H_2_O_2_ by reacting with H_2_O leading to more ^•^OH production.

#### 3.3.1. Metal Oxide-Based Graphene Composite Photocatalysts

Several metal oxide-graphene composites have been explored as photocatalysts for degradation of pharmaceuticals. Bhatia et al. [6] reported the degradation of 25 ppm of atenolol using a GO supported TiO_2_, which achieved 72% degradation under a simulated solar light irradiation for 1 h. Using Degussa P25 as catalyst, a degradation efficiency of 56% was achieved by the process. Evaluation of the effect of catalyst concentration on the system shows that while the degradation of the contaminant increased with increase in catalyst’s concentration when Degussa P25 was used, a reduction in photocatalytic activity was observed for the GO supported photocatalyst. This was attributed to the reduction in light penetration into the system as a result of the shielding effect of GO on the catalyst particles. In another study to optimize the degradation process for atenolol using a graphene-TiO_2_ composite, Bhatia, et al. [145], studied a four-factor three-level Box-Benkhen design to determine the optimal condition for the process. The photocatalyst concentration, pH, atenolol concentration and light intensity at levels of 10–20%, 4–9, 10–30 mg/L and 60–260 W/m^2^ respectively were studied. The study showed that the optimum rate constant for the process was 0.667 min^−1^, which was achieved by catalyst concentration of 10%, pH of 6.5, light intensity of 160 W/m^2^ and atenolol concentration of 30 mg/L. The effect of the presence of H_2_O_2_ on the photocatalytic degradation of acetaminophen (ACT) by a TiO_2_@rGO composite, showed that the degradation and mineralization efficiency of the catalyst could be enhanced by a multiple of 4 and 3 respectively by the introduction of H_2_O_2_ into the system [146]. Further study on the effect of rGO on the photocatalytic process showed the catalytic activity was maximum at 3% rGO concentration, with further increase resulting in inhibition of catalytic activity.

Heterojunctions based on graphene-metal oxide composite semiconductors have also been reported as photocatalysts for pharmaceutical degradation. The degradation of three pharmaceuticals: bisphenol-A (BPA), ibuprofen (IBP) and flurbiprofen (FBP) by graphene oxide based TiO_2_-ZnO heterojunction was reported by Bilgin Simsek, et al. [147]. The study showed that a degradation efficiency of 99.7, 98.5 and 98.1% of BPA, IBP and FBP was achieved by subjecting 10 mg/L of the pollutant solution to light irradiation at pH of 6.0 for 2 h under UV irradiation. The enhanced activity of the composite was attributed to the electron withdrawing properties of graphene. When other combinations between the three components of the catalyst were considered, the catalytic activity of the catalysts was reported to be in the order RGO/TiO_2_/ZnO > RGO/TiO_2_ > TiO_2_/ZnO > TiO_2_ > ZnO. Photocatalytic efficiency of Ag_2_CrO_4_/Ag/BiFeO_3_@rGO heterojunction for ciprofloxacin (CIF) degradation under broad light spectrum was reported by Kumar, et al. [148]. Apart from the advantages of Z-scheme formation, the reductive and oxidative capabilities of the composite were triggered by the plasmonic Ag^0^ electron donation-mediation and the adsorption-electron mediation of reduced graphene oxide.

#### 3.3.2. Noble Metal Based Photocatalysts

The use of noble metals such as platinum (Pt) and silver (Ag) as co-catalysts for graphene supported photocatalysts have been explored as photocatalyst for the degradation of pharmaceuticals because of their ability to enhance light absorption and improve rate of charge generation via their surface plasmon resonance (SPR) [149]. Xu, et al. [150] reported the enhanced degradation efficiency of Ag-BiOBr-rGO composite for the degradation of ketoprofen under simulated solar light irradiation. The catalytic study showed that BiOBr alone showed a degradation efficiency of 70% towards ketoprofen after 120 min, which was increased to 78% by the introduction of 5% rGO to the catalyst. Doping of Ag into the BiOBr-rGO photocatalyst at 5% mole fraction resulted in 100% degradation of the pollutant, with 65.8% mineralization efficiency. It was further observed that excess incorporation of Ag atoms into the composite resulted in decreased degradation efficiency due to electronic sinks being formed by the excess atoms. The composite also showed sufficient stability, with up to 100% efficiency still recorded after 3 cycles of usage. Similarly, Mohan, et al. [151] reported the degradation of oxytetracycline by V_2_O_5_/rGO/Pt composites. The study focused on the effect of additives such as H_2_O_2_, Na_2_CO_3_, ethanol, persulfate and NaCl on the degradation efficiency of the photocatalyst. It was reported that additives that results in radical scavenging such as NaCl and Na_2_CO_3_ inhibited the degradation of the process, while additives with potential of increasing radical generation such as persulfate and H_2_O_2_ enhanced the degradation efficiency. For H_2_O_2_, the improvement in efficiency was limited to low concentrations because H_2_O_2_ quenches the ^•^OH generated in the system at high concentration. Evaluation of the photocatalyst for real effluent treatment showed a slightly reduction in efficiency from 99% for simulated water sample to 87%.

#### 3.3.3. Metal Sulphide-Based Graphene Composite Photocatalyts

Recently, metal sulphides have been explored as semiconductor materials due to their abundance, non-toxicity and low melting points, which allows for low temperature processing [152]. The use of graphene-supported ternary sulphide composites have also been explored as photocatalysts for pharmaceutical degradation. The degradation of 4 nitrophenol and 2-nitrophenol was studied using rGO supported AgIn_5_S_8_ (rGO/AIS) as a photocatalyst [153]. The optimal rGO weight percentage in the composite was determined by photoluminescence (PL) quenching experiment, which showed that highest PL quenching was observed in the composite with 1% weight of rGO, signifying reduced electron-hole recombination. This confirmed that the lowest electron-hole recombination was observed in the 1% rGO/AIS. The photocatalytic study showed that 91% degradation efficiency was achieved by the catalyst compared to 30.5% achieved by pristine AIS for 4-nitrophenol. The efficiency of degradation was 72.5 and 93.2% respectively for the degradation of 2-nitrophenol using AIS and 1% rGO/AIS. The photocatalytic activity of photocatalysts with higher rGO weight percentage was reported to be lower compared to 1% rGO/AIS, which was attributed to the covering of the catalyst surface by excess rGO, resulting in a suppression of visible light absorption, reduction of active site formation and increased electron-hole recombination. The degradation of naproxen by rGO supported ZnIn_2_S_4_ (rGO/ZIS), showed that improved activity was achieved by rGO incorporation resulting in a degradation rate of 0.082 min^−1^, which was 1.3 times higher than the value reported for the unsupported catalyst and the degradation efficiency of the process reached 99% after 60 min [154]. Reactive species quenching experiments conducted in order to determine the active species responsible for the degradation showed that scavenging of ^•^OH from the process did not have any effect on the efficiency of the process, while scavenging of h^+^ and $O_{2}^{•\;-}$ resulted in a significant reduction in degradation efficiency.

The compositing of two or more nanomaterials with differing dimensions through covalent and noncovalent interfaces could offer unique materials with new properties for different applications [155]. By compositing 3D bismuth oxyiodide, 2D graphene oxide (GO) and 1D bismuth sulphide, Arumugam, et al. [156] reported the synthesis of Bi_2_S_3_@GO/BiOI with 2% and 1% weight composition of Bi_2_S_3_ and GO respectively for the photodegradation of tetracycline under visible light radiation. The synergistic interaction between the multidimensional components of the catalyst resulted in enhanced charge transport and separation. The optimum composition of GO was determined by evaluating the photocatalytic activity of varying amount of GO with BiOI for tetracycline degradation and the highest degradation of 82% was observed for 1% wt GO, compared to 77 and 73% degradation for 2 and 3% GO composition respectively. The 1%GO-BiOI was then coupled with varying composition of Bi_2_S_3_, with the 2% wt composition yielding the highest degradation efficiency of 98% for tetracycline degradation, compared to 88 and 89% degradation for 1 and 3% Bi_2_S_3_ composition respectively. The reaction rate constant for the photocatalyst based on differing Bi_2_S_3_ composition was 0.171. 0.350 and 0.224 min^−1^ for 1%-Bi_2_S_3_@1%-GO/BiOI, 2%-Bi_2_S_3_@1%-GO/BiOI and 3%-Bi_2_S_3_@1%-GO/BiOI respectively.

#### 3.3.4. Metal-Free Composites

Kumar, et al. [157] reported a metal free self-assembled carbon quantum dots (CQD) and rGO layers modified S@g-C_3_N_4_/B@g-C_3_N_4_ composite as visible and solar light photocatalysts for the degradation of chloramphenicol (CMP). The study showed that 99.1 and 92.4% of CMP was degraded under visible and natural solar light respectively. Combination of rGO and CQD afforded the catalyst with better adsorption capacity, enhanced visible light absorption, improved charge flow via intimate interfacial contact, reduced charge recombination and increased ROS generation. The doping of g-C_3_N_4_ with different atoms resulted in different band structures, which grew into an effective Z-scheme when combined together. The formation of heterojunctions increases the chances of $O_{2}^{•\;-}$ and ^•^OH compared to individual g-C_3_N_4_. Similarly, composites obtained from acidified g-C_3_N_4_ (ACN), biochar, polyaniline (PANI) and rGO were explored to synthesis three metal-free photocatalysts: g-C_3_N_4_/ACN/rGO@Biochar (CARB), G-C_3_N4/PANI/rGO@Biochar (CPRB) and CAN/PANI/rGO@Biochar (APRB) for degradation of ibuprofen [158]. Under Xenon lamp radiation, the APRB showed the highest efficiency of 98.4% degradation for 20 mg/L of ibuprofen after 50 min, while the efficiencies of GARB and GPRB was about 70 and 62% respectively. The reaction rate for the degradation process by APRB was 0.08025 min^−1^, which was 2.4-fold of the value obtained for GARB. Although, the three composites showed the feasibility for Z-scheme mechanism, the favourable band edge positions, reduced charge carrier recombination, higher photo-response, and ordered structure resulting in improved charge flow along interfaces accounts for the higher activity of APRB.

Table 3 presents an overview of some photocatalytic processes that employ graphene-based composite. The photocatalytic processes are the most explored application of graphene-based composites in pharmaceutical degradation. It is observed that UV light source are still the most explored energy source for photocatalytic process, with only few studies employing visible/solar light sources. This shows the need for improved band gap tuning techniques which could enhance the utilization of visible/solar light sources, leading to an improvement in the economic visibility of this process. The wide range of pharmaceuticals that have been studied, the short reaction time and the high degradation efficiency of this process as presented in the table, is a proof of the great potential of this process for removal of pharmaceuticals from the environment.

### 3.4. Electrocatalytic Process

The Fenton process is one of the prominent AOPs for the degradation of contaminants. The process involves the generation of reactive ^•^OH radicals by the reaction of hydrogen peroxide with ferrous iron catalyst (Equations (3) and (4)). The formation of large ferric hydroxide sludge, narrow pH requirement, large Fenton reagent consumption and high risk involved in production, transportation and storage of H_2_O_2_ are serious drawbacks for the process despite its high potential [180].

(3) $${\mathrm{Fe}}^{2+}+{\;H}_{2}O_{2}\to {\mathrm{Fe}}^{3+}+{\;\mathrm{HO}}^{-}+{}^{•}OH$$

(4) $${\mathrm{Fe}}^{3+}{\;H}_{2}O_{2}\to {\mathrm{Fe}}^{2+}{\;\mathrm{HO}}_{2}^{•}+H^{+}$$

With the electro-Fenton process (EF), H_2_O_2_ for the Fenton process is electrogenerated in the reaction system via the two-electron oxygen reduction reaction at the cathode (Equation (5)). Thus, circumventing the challenges associated with H_2_O_2_ addition in the Fenton process [181]. The continuous reduction of Fe^3+^ to Fe^2+^ at the surface of the cathode via the direct one electron reduction or by reaction with H_2_O_2_ (Equation (4)) is the main driving force behind the EF process [182]. The EF process has gained much attention due to its high efficiency, low cost and facile operating conditions [183,184]. Also, advances in electro-Fenton electrode development has led to composite electrodes into which solid Fenton catalysts are immobilized such as ferrite-carbon aerogel [185], Fe_3_O_4_@carbon fiber paper@polyaniline [186], Fe_3_O_4_@Fe_2_O_3_/activated carbon aerogel [187]. These functionalized cathodes have the potential of achieving the Fenton reaction over a wide pH range.

(5) $$O_{2}+2H^{+}+2e^{-}\to H_{2}O_{2}$$

(6) $${\mathrm{Fe}}^{3+}+{\;e}^{-}\to {\mathrm{Fe}}^{2+}$$

The degradation of acetaminophen in an electro-Fenton process employing heteroatom-doped graphene aerogel cathode and carbon-magnetite catalyst, showed complete degradation was achieved in 240 min, while 45.5% mineralization efficiency was achieved in 360 min [188]. The degradation efficiency was influenced by the amount and nature of heteroatom, while the mineralization efficiency was strongly correlated to the amount of iron leached from the catalyst. The degradation efficiency of homogeneous and heterogeneous electro-Fenton process using FeSO_4_•7H_2_O and graphene oxide-Fe_3_O_4_ composite respectively as catalyst for the degradation of chloramphenicol and metronidazole was reported by Görmez, et al. [189]. The study showed that 57 and 71% mineralization was obtained for the degradation of 80 g/L^−1^ metronidazole and chloramphenicol after 300 min for the homogenous process. Under the same reaction condition, the graphene oxide-Fe_3_O_4_ heterogeneous catalyst achieved 73 and 86% mineralization efficiency for metronidazole and chloramphenicol respectively, with degradation efficiency reaching above 99%. The process was observed to be greatly influenced by the pH of the system, with optimum pH being 3. This was attributed to the positive charge induced on the surface of the catalyst at low pH, thus favouring an electrostatic interaction with the pollutant molecules which were also present in their ionic form.

**Figure 10:**
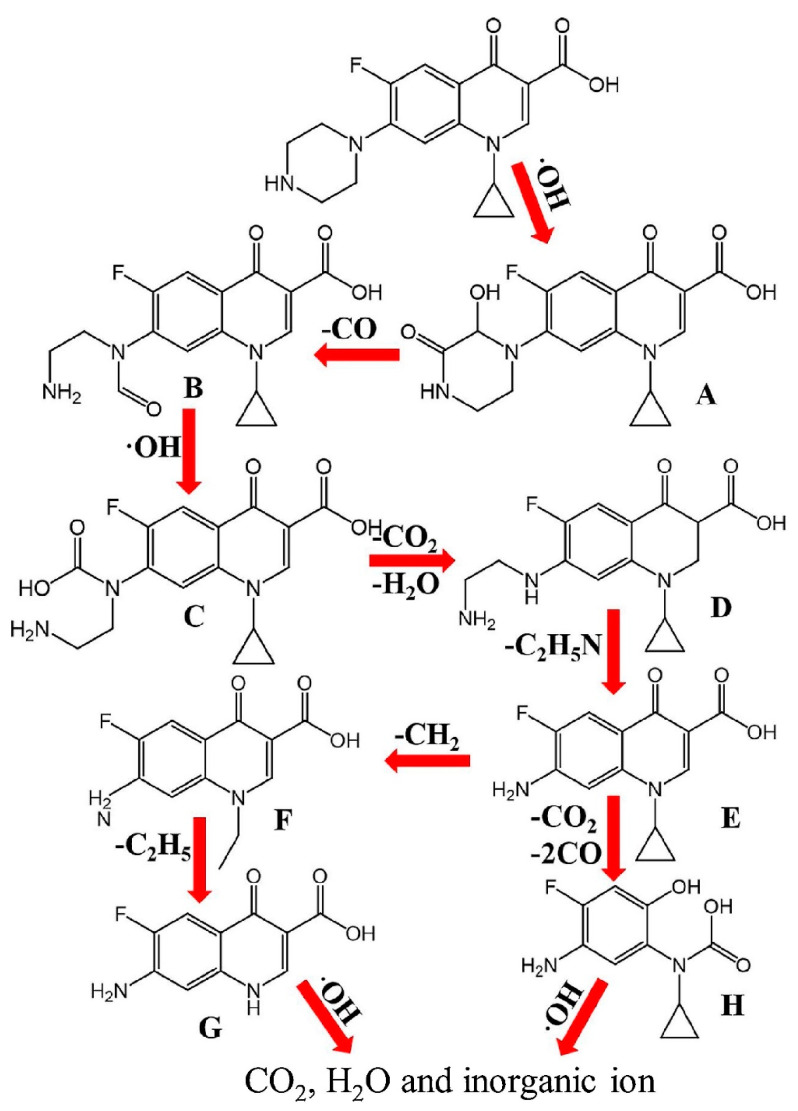
Mechanism of degradation of ciprofloxacin in an RGO-Ce/WO_3_ nanosheets modified carbon felt cathode based Electro-Fenton process. Reprinted with permission from Mi, et al. [191]. Copyright (2019) Elsevier.

A novel γ-FeOOH graphene polyacrylamide carbonized aerogel was reported as air-cathode for the degradation of sulfamethoxazole (SMX) [190]. The study showed a gradual increase in the conductivity, and specific surface area of the cathode material until an optimum concentration of 5 mg/mL^−1^ GO was reached. Above this optimum GO concentration, a notable decrease in surface area was observed despite the increase in conductivity. This was attributed to the reduced incorporation of GO into the hydrogel when excess GO was employed. When the catalytic activity of the catalyst was evaluated using 0.15 g of the catalyst on 500 mL of 0.1 mM SMX at 200 mA, a complete degradation was achieved after about 120 min with a degradation rate constant of 0.51 × 10^−1^ min^−1^. In another study, Mi, et al. [191], reported the mechanism of degradation of ciprofloxacin (CIP) by RGO-Ce/WO_3_ nanosheets modified carbon felt cathode. It was observed that the degradation process progressed by the oxidative degradation of the piperazine ring and oxidative cleavage of the cyclopropane ring of CIP. As shown in Figure 10, the oxidative degradation process was achieved via hydroxylation, decarboxylation and N-dealkylation processes.

Aside from electro-Fenton processes, graphene-based composites have also been explored as anodic membranes for the electrochemical oxidation of pharmaceuticals. The excellent electrical conductivity of graphene and electrooxidation capacity of SnO_2_ were explored in a G/SnO_2_/CFs composite for the electrochemical degradation of SMX [192]. The membrane showed improved degradation efficiency of 85% and high stability, withstanding 10 cycles without any significance loss of activity. Mechanism study showed that two transformation pathways: hydroxyl radical attack and ring cleavage were the identified routes for the degradation process. Presented in Table 3, are the process overview of other electro-Fenton process employing graphene-based composites are presented in Table 4 and the potential application of graphene-based composites as catalysts, electrodes or membranes in this process, shows the significant role these composites can play in the further development of these AOP technique.

### 3.5. Sonocatalytic/Sono-Photocatalytic Process

Different studies have shown that by combining ultrasonic irradiation with other AOPs such as ultrasonic/photocatalysis, ultrasonic/Fenton process and ultrasonic/catalysis, the efficiency of contaminant sdegradation could be enhanced. Coupling of ultrasonication with other AOPs often leads to: (i) an increase in mass transfer of pollutants between catalysts surface and liquid phase, (ii) enhancement of reactive radical generation, and (iii) de-aggregation of the nanocomposite particles. The sonocatalytic degradation of sulfasalazine and diclofenac by Ag_2_O/CdO/CeO_2_/rGO was reported by Mirzazadeh and Lashanizadegan [197] with efficiencies of 100% and ~85% reported for sulfasalazine and diclofenac respectively. The efficiency of the process was significantly influenced by the weight percent of Ag in the composite and the optimum composition of Ag was reported to be 5%. Kinetic studies showed a reaction rate constant of 0.11 and 0.018 min^−1^ for sulfasalazine and diclofenac respectively. The effect of sonolytic frequency on the degradation of carbamazepine and acetaminophen by GO/β-Bi_2_O_3_/TiO_2_/Bi_2_Ti_2_O/heterojunction was reported by Lee, et al. [198]. Although, increasing the frequency leads to the enhancement of acoustic cavitation and subsequently the catalytic activity, increasing the frequency beyond an optimal level resulted in reduced activity due to the reduction in the occurrence of collapsing events, which may result to reduced number of small cavity bubbles. Comparing degradation at frequencies of 28, 580 and 970 kHz, the process at 580 kHZ was found to be most effective for carbamazepine and acetaminophen degradation.

Moradi, et al. [199] reported the sonophotocatalytic degradation of sulfamethoxazole using MgO/ZnO/Graphene nanocomposite. Incorporation of graphene and ZnO with MgO enhanced its catalytic activity under both UV and visible light radiation. Complete degradation of SMX was achieved after 120 min. The ultrasound played an important role in ensuring clean-up of the catalyst surface and jointly enhanced the production of reactive radicals with the light source. The photocatalytic interaction between the composite and light source was also significant in enhancing the efficiency of the process.

## 4. Factors Influencing the Degradation of Pharmaceuticals

Generally, the efficiency of any advanced oxidation process is significantly influenced by factors that may influence the reactive radical generation. These factors are often referred to as controlling parameters and the most studied parameters include concentration of the pharmaceutical, catalyst concentration, dissolved oxygen levels, pH, concentration of oxidants, water matrix, light source and intensity.

### 4.1. Concentration of Pharmaceuticals

High initial concentration of pollutants usually lead to a higher rate of adsorption on the catalyst surface. However, a commensurate increase in reactive radical generation is usually needed to sustain the degradation process. Therefore, increasing the initial concentration of the pollutant usually result in increased degradation burden on a unit catalyst due to limitation in the amount of reactive species generated in the process. Also, the coverage of the catalyst surface by the adsorbed molecules have also been suggested to be capable of blocking the catalyst surface from receiving sufficient light energy for generation of reactive radical species in photon-based processes [200]. Therefore, Increasing the concentration of pollutant has been found to generally result in process efficiency reduction.

### 4.2. Effect of pH

It is well established that the adsorption and surface charge of a catalyst is greatly influenced by the system pH. Also, the ionic form of the pollutant in solution is significantly influenced. At pH value equal to the point of zero charge (pH_pzc_) of the catalyst, surface charge is usually zero, while pH values lower or higher than the pH_pzc_ leaves a net positive or negative charge on the catalyst [201]. Kaur, et al. [202] confirmed this trend in the study of the effect of pH on the degradation of triclosan using rGO-TiO_2_ composite. At a pH value below the pH_pzc_ of the catalyst complete degradation was achieved, while the degradation was significantly suppressed at higher pH. In the degradation of amoxillin by graphene-titanium oxide nanotubes composites, Song, et al. [203] reported a gradual increase in degradation efficiency with increase in pH from 3 to 9; however, increasing the pH to11 resulted in a decrease in efficiency due to the similar charge on the catalyst and amoxicillin molecule. Therefore, depending on the ionic state of the pollutant and the net charge on the catalyst surface, the adsorption of the pollutant which is significant for the degradation process needs to be altered towards the enhancement of the degradation process.

### 4.3. Effect of Catalyst Concentration

Effect of catalyst concentration on the degradation efficiency usually varies for photon-based processes and non-photo-based processes. While in general, the increase in the catalyst concentration increases the quantity of reactive radical species generated in a system, in photon-based processes, increasing the catalyst concentration beyond certain concentration level is found to reduce the degradation efficiency. This is due to reduced light peneteration into the reaction system, leading to reduced radical generation. In the study by Sayadi, et al. [175], increasing the catalyst concentration from 0.25 g/L to 0.75 g/L led to an increase in degradation efficiency. However, beyond this concentration level, a reduction in catalytic activity was observed due to increased turbidity of the solution, which reduces light penetration and dispersion.

### 4.4. Dissolved Oxygen

Dissolved oxygen (DO) in a catalytic system acts as an electron acceptor, thus reducing the rate of electron-hole recombination, which enhances the degradation efficiency of a process. Also, dissolved oxygen could help in radical intermediate stabilization; aromatic ring cleavage induction and mineralization [204,205]. Azim, et al. [206] reported an increase in the catalytic activity of GO as the level of DO in the system was increased. However, Lee, et al. [207] observed that in processes that employed DO generator in order to improve level of DO, bubbles formed by the generator got attached to the catalyst surface, thereby reducing the efficiency of the process. At high DO concentration level, this effect was mitigated and enhanced catalytic activity was observed. Subramanian and Kannan [208], reported that the level of DO in a system is important for processes involving high light intensities due to the large quantities of holes and electrons generated, as the electron scavenging role of dissolved oxygen becomes significant.

### 4.5. Light Intensity

Light intensity is an important factor to be optimized for a photocatalytic process as it contirbutes significantly to the overall cost of a process. Thus, its effective use is a major factor to be considered in the design and operation of a process. In terms of the effect of light intensity on reaction rate, three types of relationships have been identified. A linear relationship is generally observed between degradation rate and light intensity, when low intensity light is used. At high light intensity, the degradation rate is independent of the light intensity. For intermediate light intensities, the power-law between light intensity and reaction rate holds with an exponent varying between 0.5–0.8 [209]. Malekshoar, et al. [209], studied the effect of light intensity on the activity of Graphene-TiO_2_ composite on degradation of phenol, by varying the light intensity between 20–10 mW/cm^2^. The authours observed a linear relationship between the intensity and reaction rate at light intensities below 50 mW/cm^2^, while at higher intensities the rate increased with a power of 0.7.

### 4.6. Water Matrix

To determine the suitability of any AOP for wastewater treatment, it is important to evaluate the effect of co-existing susbstances such as organic acids (e.g., acetic and formic acids), natural organic matters (NOM) (e.g., fulvic and humic acids) and inorganic ions (e.g., Cl-, ${\mathrm{NO}}_{3}^{-}$, ${\mathrm{CO}}_{3}^{2-}$ and ${\mathrm{SO}}_{4}^{2-}$ on the removal efficiency of pollutants. The water matrix also significantly influence the optimal condition for any degradation process. The presence of co-existing substances could either have a negative or positive influence on the degradation efficiency of a process. The negative influence of co-existing substances may arise from their undesirable reactions with generated radical species and the light scattering ability of some suspendeded solids. Meanwhile, the positive effect of co-existing substances arises due to the oxidation and reduction properties of some of these compounds, which could serve either as oxidants or reductants in the catalytic process. Furthermore, compounds with carboxylic and hydroxy groups could form complexes with high quantum yield, leading to enhanced photo-reduction reaction [210,211,212]. Tokumura, et al. [212] explored the effect of water matrix on the degradation of carbamazepine and diclofenac using three AOPs: photo-Fenton, photocatalysis and combined oxine and hydrogen peroxide oxidation process. The authors reported an increase in the efficiency of the Fenton process due to additional iron ions from the water matrix. However, the photo-reduction reaction was inhibited by the light scattering effect and complex-formation of co-existing substances. For the photocatalytic and combined ozone and hydrogen peroxide oxdiation process, the scavenging effect of the co-existing substances was a main inhibiting factor to the efficiency of the processes.

### 4.7. Comparative Analysis of Graphene-Based Catalytic Process for Degradation of Pharmaceuticals

The comparative analysis of the identified degradation techniques for degradation of pharmaceuticals by graphene-based catalytic processes, was carried out in terms of efficiency, reaction rate, mineralization rate and the time required to reach 90% degradation. The result of the analysis is shown in Table 5. All the five degradation techniques showed very close degradation efficiency above 90%, which confirms the potential of these techniques in pharmaceutical degradation. In terms of the reaction rate for each process, the chemical oxidation process showed the highest average reaction rate, with photocatalysis and electrocatalysis showing very similar degradation rates. The direct degradation and sonocatalytic/sonophotocatalytic processes showed low degradation rates. Comparing the average mineralization of each process, it is observed that electrocatalysis and sonocatalytic processes had the highest potential for conversion of pharmaceuticals to CO_2_ and water.

The time required to attain 90% degradation for each process was evaluated using the equation:

(7) $$t_{0.9}=\;\frac{2.3035851}{k}$$

where *k* is the reaction rate constant for each of the study. The average of the *t*_0.9_ for each process, showed that photocatalytic process had the lowest required time to achieve 90% degradation compared to other processes. This was followed by electrocatalysis and chemical oxidation processes.

## 5. Future Perspectives

The excellent catalytic activity of graphene-based composites for pharmaceutical degradation has been discussed in this review. In many of the explored studies, graphene/graphene derivatives have been used as a minor component of the catalytic materials. However, few studies have shown that composites constituting graphene/graphene oxide as major components have also shown good catalytic activity. It becomes important for the optimal graphene/graphene derivative to be explored and important factors such as cost, sustainability, efficiency in determining the preferred constitution. Also, discussed in this review were mainly bench scale studies with limited studies available for pilot scale studies. With the large volume of bench scale results obtained so far, studies on graphene-based composites for pharmaceutical degradation seems ripe for pilot scale studies, which might set it on the part to full commercialization.

## 6. Conclusions

A review of processes employing graphene-based composites as catalysts for degradation of pharmaceuticals have been presented in this study and five different degradation processes—direct catalytic degradation, chemical oxidation process, photocatalysis, electrocatalytic processes and sonocatalytic/sono-photocatalytic processes—have been identified in the explored literatures. Each of the process showed high degradation efficiencies, with the generation of reactive radical species being responsible for the activity of each process. Incorporation of graphene/graphene derivatives, was observed to enhance the degradation efficiency by improving on the generation of radical species, through improved surface area, light absorption and reduce recombination of generated charge carriers. A key factor in enhancing the activity of the composite is the determination of the optimal weight percentage of graphene required for improving the catalytic activity. Comparative analysis of these techniques showed that while they exhibit similar degradation efficiency, the photocatalytic process requires the least amount of time to attain 90% degradation, while the electrocatalytic and sonocatalytic processes are the most desirable in terms of mineralization potential.

## Figures and Tables

**Table 1:** Some therapeutic classes of pharmaceuticals, toxicity level and their level of detection in the environment.

Therapeutic Class	Example	Structure	Acute Toxicity Level/Test Organism	Detected Level in the Environment	Matrix/Country	Ref.
Anti-inflammatories	Diclofenac	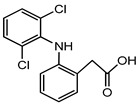	90 μg/L; fish	0.44–7.1 μg/L	Municipality treatment plant	[24,25]
Antibiotics	Ciprofloxacin	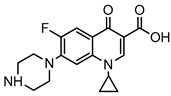	84–1000 mg/L; fish	6.5–31 mg/L	WWTPs/India	[26]
Antiepileptics	Carbamazepine	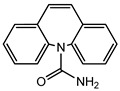	0.01 μg/L; invertebrate	425–3500 ng/L	Ground water/UK	[27,28]
Lipid regulator	Propranolol	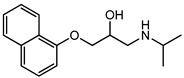	1.3–1.7 mg/L; invertebrate	20–92 ng/L	WWTPs/Canada	[29,30]
Hormones	17*β*-Estradiol	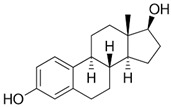	1500 μg/L; invertebrate	3–11 ng/L	Surface water/China	[31,32]
Anticancer	Tamoxifen	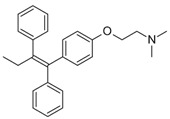	-	27–212 ng/L	Surface water/UK	[33]
Cardiovascular	Metoprolol	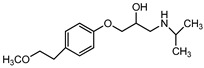	43.0–47.4 mg/L; *Ceriodaphnia dubia*	20–116 ng/L	Surface water/Hungry	[34]
Antidiabetics	Metformin	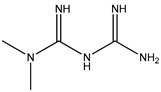	--	100–1700 ng/L	Surface water/Germany	[35]

**Table 2:** Chemical oxidation processes based on graphene-based composites for degradation of pharmaceuticals.

Catalyst	Pharmaceutical	Process Parameter	Degradation Efficiency	Reaction Rate	Process Overview	Ref
graphene	Diclofenac, Norfloxacin, Tetracycline, sulfamethoxazole	|Cat| = 100 mg/mL; |Pol| =5 mg/L; |PMS| 0.5 mM pH = 4.0; Time = 120 min	84 100 100 100	5.92 × 10^3^ min^−1^	The effect of adsorption on removal of pharmaceuticals was studies. It was observed that degradation was influenced by the adsorption capacity of graphene for the pharmaceuticals	[136]
CoFe_2_O_4_-GO	Norfloxacin	|Cat| = 0.3g/L; |Pol| =15 μM; |PMS| = 0.5 mM; pH = 7; Time = 20 min	100	0.29 min^−1^	The study reported that the compositing of CoFe_2_O_4_ with graphen oxide (GO) altered the mechanism of norfloxacin which was dominated by non-radical processes, unlike in degradation involving pristine CoFe_2_O_4_ which was dominated by radical processes	[137]
TiO_2_/rGO/PS	Antipyrine	|Cat| = 150 mg/mL; |Pol| =5 mg/L; |PMS| = 300 mg/L mM pH = 7.0; Time = 45 min	100	-	The reaction between the conduction band electrons and persulfate reduced the recombination of the generated e-/h+ pairs leading to enhanced degradation of pollutant	[138]
rGO-Ag^0^/Fe_3_O_4_	Phenol, acetaminophen, ibuprofen, naproxen, bisphenol	|Cat| = 1 mg/mL; |Pol| =10 μM; |PDS| = 1 mM pH = 7.0; Time = 360 min	50 99 >90 <50 <50	0.46 h^−1^ 6.19 h^−1^ 1.93 h^−1^ 0.90 h^−1^ 0.57 h^−1^	Under acidic condition (pH 4) the catalyst showed low substrate-dependent activity, however under neutral/basic conditions (pH 7 and 10), the activity of the catalyst was substrate specific.	[139]
Go-MCM-Fe	Levofloxacin hydrochloride	Cat| = 50 mg/mL; |Pol| =100 mg/L; |$S_{2}O_{8}^{2-}$| = 0.02 g pH = 4.3; Time = 60 min	97.1	0.354 min^−1^	The solvothermal process was used in compositing MCM-41, GO and iron. The incorporation of GO resulted in increased strength of the composite. Also, increasing the quantities of GO and iron lead to increased catalytic activity. Quenching experiments confirmed ^•^OH and ${\mathrm{SO}}_{4}^{•-}$ radicals as the main ROS generated in the system	[140]
N-rGO/Fe_3_O_4_/PS	Norfloxacin	Cat| = 100 mg/mL; |Pol| =20 mg/L; |$S_{2}O_{8}^{2-}$| = 10 mM pH = 7.0; Time = 210 min	100	0.0047 min^−1^	The prepared composite showed high adsorption capacity for norfloxacin, which was degraded by ^•^OH and $SO_{4}^{•-}$ generated by the activation of persulfate	[141]
Ni@NPG	Sulfachloropyridazine	Cat| = 0.05 g/mL; |Pol| =20 mg/L; |$S_{2}O_{8}^{2-}$| = 2 g/L; Time = 30 min	100	0.46 min^−1^	The synthesized N doped graphene encapsulated Ni composite benefited from the 3-dimensional structure of the composite, and the synergistic effect of N-doping and nickel encapsulation. The degradation mechanism revealed a dominant non-radical pathway.	[142]
GO-TiO_2_	Diclofenac	Cat| = 0.1 g/L; |Pol| =20 mg/L; |$S_{2}O_{8}^{2-}$| = 10 g/L pH = 5.4; Time = 14 min	93.06	-	The degradation of diclofenac was studied using composite centered design. The most significant variable that influences the degradation efficiency was pH and the catalyst concentration	[143]

**Table 3:** Photocatalytic degradation of pharmaceuticals using graphene-based composites.

Graphene Composite	Light Source	Pollutant	Process Parameters	Degradation Efficiency	Reaction Rate	Process Description	Ref.
ZnSnO_3_/RGO	Mercury lamp 500 W	Metronidazole	|Pol| = 5 mg/L; |Cat| = 0.2 g; Time = 180 min	72.5%	-	Photocatalytic activity of the catalyst was reportedly higher under UV light radiation, which achieved complete degradation after 20 min. compare to visible light radiation.	[159]
AgFeO_2_/G@Cu_2_(BTC)_3_	Halogen lamp; 500 W	Diclofenac, amoxicillin	|Pol| = 5 g/L; Time = 150 min;	91.4 92.9	8.7 × 10^−2^ min^−1^ 6.5 × 10^−2^ min^−1^	Improved photocatalytic activities was attributed to enhanced charge transfer mechanism through direct Z-scheme from the Cu_2_(BTC)_3_ conduction band to the AgFeO_2_ valence band, with graphene acting as solid electron mediator/acceptor	[160]
ZnONP/GONS	UV lamp (254 nm; 1012 μW/cm^−2^)	Levofloxacin	|Cat| = 0.4 g/L; |Pol| = 40 μg/mL; pH = 9.0; Temp. = 298 K; Time = 120 min	99.2	-	A mixed fractional factorial designed was employed in studying the effectiveness of combining ZnO nanoparticles with graphene oxide nanosheet. Factors such as pH, type and concentration of nanoparticles, initial drug concentration and exposure time. All factors showed significant effect of degradation efficiency except pH and exposure time.	[161]
V_2_O_5_/rGO/Pt	Xenon lamp; 150 W	Oxytetracycline	|Cat| = 0.5 g/L; |Pol| = 50 μg/mL; pH = 9.0; Temp. = 298 K; Time = 60 min	98	1.87 × 10^−2^ min^−1^	Photocatalytic activity of V_2_O_5_ was enhanced by compositing with rGO and Pt. Compositing with rGO resulted in increased efficiency of 90% compare to 58% observed for bare V_2_O_5_. The photocatalytic activity was further increased to 98% by further doping of the rGO/V_2_O_5_ with 1% Pt.	[162]
GO-ZnWO_4_	UV light	Cetirizine hydrochloride	|Cat| = 425 mg/L; |Pol| = 10 mg/L; pH = 7.0; Time = 120 min	89	-	The photocatalytic activity of GO-ZnWO_4_ for cetirizine hydrochloride degradation was optimized via a response surface methodology. The model obtained for the process showed that factors such as pollutant concentration, catalyst dose, pH and irradiation time influenced the degradation process with interactions effects also existing between all the process parameters	[163]
TiO_2_/Fe_2_O_3_/GO	UV lamp (15 W; 254 nm wavelenght)	Metronidazole	|Cat| = 1 g/L; |Pol| = 10 mg/L; pH = 5.0; Time = 120 min	97	-	The effect of mineral salts such as NaCl, CaCl_2_, KcL, NaHCO_3_, MgSO_4_ and Na_2_SO_4_ on the degradation efficiency of the catalyst was explored at concentration range of 50-800 mg/L. The efficiency of the process decreased with increase in mineral salt concentration for Na_2_SO_4_, MgSO_4_ and NaHCO_3_ resulting in 42.43, 38.08 and 37.73 efficiency reduction respectively. For minerals such as NaCl and KCl, efficiency was reduced up to concentration level of 200 mg/L, after which an upward trend in photocatalytic activity was reported. The effects of these mineral salts were correlated with their influence on the pH of the process.	[164]
Pt-rGO-TiO_2_	Low pressure mercury vapour UV lamp (254 nm, 8W) and simulated light	Propranolol	Cat| = 0.4 mg/mL; |Pol| = 0.05 mmol; pH = 5.0; Time = 120 min	79% under UV irradiation and 94% under simulated solar light	-	The efficiency of the catalyst for propranolol degradation was observed to be higher under simulated solar light (94%) compare to UV light (79%). By rational design of the ternary heterostructure, with good efficiency in the UV spectrum and with renewable solar energy can be obtained	[165]
Ag/AgBr/GO	Direct solar radiation	Diclofenac	Cat| = 1.0 g/L; |Pol| = 25 mg/L; pH = 7.0; Time = 6 min	95	-	A facile ultrasonic irradiation-based processes was explored in the synthesis of the photocatalyst. The incorporation of GO into the catalyst enhanced the photocatalytic activity of the composite by improving the adsorptive capacity of the catalyst via intermolecular **ℼ**-**ℼ** interaction with diclofenac. Improved charge transport and reduced charge carrier recombination were other effects of GO in the catalyst.	[166]
GO-P25	Medium-pressure mercury vapour lamp (50 mW/cm^2^; 350 nm)	Diphenhydramine	Cat| = 1.0 g/L; |Pol| = 3.40 × 10^−4^ mol/L; pH = 7.0; Time = 60 min	90	54 × 10^−3^ min^−1^	Composites were prepared by a simple mixing and sonication method. Varying the GO content and heat-treatment under nitrogen altered the activity of the photocatalyst. Activity of the catalyst was enhanced by GO reduction and the high contact between TiO_2_ and carbon phase	[167]
rGO-TiO_2_	For UV/Vis process: Medium-pressure mercury vapour lamp (50 mW/cm^2^; 350 nm). For visible light process: Cut-off long pass filter (λ = 430 nm; 6 mW/cm^2^)	Diphenhydramine	Cat| = 15 mg; |Pol| = 3.40 × 10^−4^ mol/L; pH = 7.0; Time = 60 min	100 under UV/Vis irradiation 40% under Visible light radiation	62.3 × 10^−3^ min^−1^ 3.4 × 10^−3^ min^−1^	Under both near-UV/Vis and visible light irradiation, the activity of the catalyst was composition dependent. Under UV/Vis irradiation photogenerated holes were the primary active species, while under visible light radiation, radical mediated oxidation of the pollutant was observed	[168]
Ag_2_O/ZnO/rGO	500 W xenon lamp	Bisphenol A	Cat| = 0.8 g/L mg; |Pol| =10 mg/L; Time = 180 min	80%	0.084 min^−1^	The photocatalyst was prepared through a rapid hydrothermal process. The band gap of ZnO was reduced by Ag doping, while rGO reduced the electron-hole pair recombination. Optimum doping of 5% and 3% percent was reported for Ag and rGO respectively	[169]
Graphene_SBA/TiO_2_	Xenon lamp	Tetracycline	Cat| = 50 μg; |Pol| =30 μg/L; Time = 60 min	89%	0.0367 min^-1^	The composite exhibited high activity dye to increased adsorption of the pollutant via ℼ-ℼ interaction. The SBA/TiO2 acted as a medium for migration of the photogenerated carriers, while the graphene sheet increased the number of active site	[170]
rGO/GNW hydrogel	UV lamp (24 W, wavelength of 254 nm)	Ethenzamide	Cat| = 10 mg/; |Pol| = 500 ppb; Time = 120 min	99.0 (under UV light)	1.014 min^−1^	Graphene/TiO_2_ nanowire was implanted into graphene hydrogel to obtained a 3D network with enhanced catalytic activity under VUV, UV and Vis-NIR light irradiation. The hydrogel was effectual in overcoming the mass transfer resistance due to the low concentration of the pollutant	[171]
N-TiO_2_/GR	50 W mercury lamp (365 nm)	Mixture of oxytetracycline chlortetracycline, sulfamethazine, norfloxacin	|Cat| = -; |Pol| =30 mg/L; pH = 11 Time = 160 min	65% 55 45 65	0.0063 min^−1^ 0.0053 min^−1^ 0.0032 min^−1^ 0.0052 min^−1^	N-TiO_2_/Gr with large surface area was prepared by depositing monolayer graphene on N-TiO_2_ thin film. The catalyst showed enhanced photocatalytic activity towards the degradation of a mixture of pharmaceuticals	[172]
Ag_3_PO_4_/graphene oxide	White LED lamps (12-W)	Ketoconazole	|Cat| = 1.62 g/L; |Pol| =5.87 mg/L; pH = 8; Time = 93.34 min	96.53	0.024 min^−1^	The photocatalytic activity of the Z-scheme Ag_3_PO_4_/GO was studied using the central composite design for modeling and optimization. The predicted optimum degradation correlated well with the experimental degradation carried out at the predicted conditions	[173]
G/A/TNS	Simulated visible light (Xenon lamp 450 W, radiation intensity- 90 mW/cm^2^	Sulfamethazine	|Cat| = 0.8 g/L; |Pol| =5 mg/L; pH = 8; Time = 240 min	96.1	0.493 h^−1^	The incorporation of graphene into the composite resulted in band gap reduction from 3.1 to 2.8 eV at the optimum graphene concentration of 0.5 wt%	[174]
GO@Fe_3_O_4_/ZnO/SnO_2_	UV-C irradiation (6W lamps)	Azithromycin	Cat| = 1 g/L; |Pol| =30 mg/L; pH = 3; Time = 120 min	90.06	0.027 min^−1^	The catalytic activity of the composite was evaluated for both batch and continuous processes. The breakthrough time was influenced by the bed height, flow rate and the initial concentration of the pollutant.	[175]
rGO/Fe_3_O_4_	Direct solar light (intensity = 850 W/m^2^)	Carbamazepine	Cat| = 1 g/L; |Pol| =30 mg/L; Time = 180 min; pH = 6.5	90%	0.0021 min^−1^	The optimum incorporation of reduced graphene oxide lead to improve surface area of the composite. Enhanced ^•^OH radical and holes generation accounted for the improved activity	[176]
BVO/rGO	Fluorescent lamp (55 W; 550 nm wavelength)	Tetracycline	|Cat| = 45 mg /L; |Pol| =25 mg/L; Time = 85 min; pH = 10.5	99	-	A bismuth vanadate (BVO) composited with rGO was synthesized via a facile process. The catalyst showed a degradation efficiency strongly influenced by the pH of the process.	[177]
Fe_3_O_4_@rGO@ZnO/Ag-NPs	LEDs (light intensity = 12 μW/cm^2^ and 365 nm emission peak Tungsten xenon lamp (300 W)	Metformin	|Cat| = 40 mg /L; |Pol| =20 mg/L; Time = 60 min; pH = 5.4	100 100	0.065 min^−1^ 0.055 min^−1^	The catalytic activity of the composite was improved by the synergistic improvement of light absorption and adsorption capacity. The magnetic nature of the composite also made separation easier to achieve.	[178]
ZnFe_2_O_4_-GR	Xenon lamp (300 W)	Paracetamol	|Cat| = 1 mg /L; |Pol| =10 mg/L; Time = 180 min	97.4	-	A nano-hybrid with photocatalytic activity that increased with weight percentage of graphene was synthesized though ZnFe_2_O_4_ is photo-catalytically inactive under visible light radiation. The optimal graphene concentration was at 4w% of the catalyst composition	[179]

**Table 4:** Fenton processes based on graphene composites for degradation of pharmaceuticals.

Graphene Composite	Role	Pollutant	Process Parameters	Amount of H_2_O_2_ Generated (mg/h/cm^2^)	Efficiency	Reaction Rate	Ref.
3D-GN@nZVI	Catalyst	Sulfadiazine	|Cat| = 0.5 g/L; |Pol| = 10 mg/L; pH = 3.0; Temp. = 298 K; Time = 120 min	-	81	-	[193]
Fc-ErGO	Cathode	Ciprofloxacin	|Pol| = 10 mg/L; pH = 7.0; Temp. = 298 K; Time = 180 min	-	99	0.035 min^−1^	[194]
3D-graphene	Cathode	Nalidixic acid	|Pol| = 15 mg/L; pH = 3.5; Current intensity = 300 mA Temp. = 298 K; Time = 300 min	28.19	90	-	[195]
Fc-ErGO	Rotating disc electrode	Ciprofloxacin, carbamazepine	|Pol| = 10 mg/L; pH = 3.0; Current intensity = 1.5 VTemp. = 298 K; Time = 60 min	175 mg/L	99.9 99.9	0.199 min^−1^ 0.082 min^−1^	[182]
Cu-rGO	electrode	Diclofenac	|Pol| = 0.2–200 mg/L; pH = 5.0–11.0; Voltage = −0.5–1.0 V; Time = 60 min	-	100	2.2–8.1 × 10^−3^	[196]

**Table 5:** Comparative analysis of graphene-based composite techniques for pharmaceutical degradation.

	Average Process Efficiency (%)	Average Reaction Rate (min^−1^)	Average Mineralization (%)	Average Time to Achieve 90% Degradation (min)
Direct degradation process	92.0	0.002	-	1123
Chemical oxidation process	94.5	0.321	65	69.2
Photocatalysis	95.1	0.177	63.15	28.4
Electrocatalysis	91.9	0.170	81.0	56.5
Sonocatalytic/sono-photocatalytic process	92.5	0.048	81	97.6

## Data Availability

Not available.
